# Penta-Helix Model of E-Government in Combating Corruption in Indonesia and Malaysia: The Moderating Effect of Religiosity

**DOI:** 10.12688/f1000research.121746.3

**Published:** 2022-11-29

**Authors:** Pupung Purnamasari, Noor Afza Amran, A. Harits Nu'man, Rusman Frendika, Mohamad Naimi Mohamad Nor, Mohamad Sharofi Ismail

**Affiliations:** 1 Faculty of Economic and Business, Universitas Islam Bandung, Bandung, West Java, 40116, Indonesia; 2 ASIAN Research Institute for Corporate Governance, Universiti Utara Malaysia, Sintok, Kedah, Darul Aman, 06010, Malaysia; 3 Faculty of Engineering, Universitas Islam Bandung, Bandung, West Java, 40116, Indonesia; 4 Tunku Puteri Intan Safinaz School of Accountancy, Universiti Utara Malaysia, Sintok, Kedah, Darul Aman, 06010, Malaysia

**Keywords:** Anti-corruption, E-Government, Information technology, Penta-helix, Religiosity

## Abstract

**Background**: E-government is an initiative taken by governments worldwide to align the administration of their countries. Governments have utilized the internet as part of a transition into a globalized economy. This helps reduce red tape and procedures in dealing with people in government agencies. This study aims to develop an e-government model as an anti-corruption strategy by applying the Penta-helix model and religiosity as the moderating variable.

**Methods**: The data was gathered from government officials, representatives in business, media, academia, and NGOs, in Indonesia and Malaysia in 2021. Online questionnaires were distributed to 240 respondents from Indonesia and Malaysia. In addition, SPSS v.25 and SEM AMOS v.25 were used to analyze the data.

**Results**: The findings indicate that the Penta-helix elements and religiosity could help to reduce corruption in Indonesia. Meanwhile, Malaysia must increase its human resource competency and embed the religiosity element as a tool to reduce corruption.

**Conclusion**: Penta-helix and religious factors should be incorporated by organizations in Malaysia and Indonesia as part of their strategy in combating corruption.

## Introduction

Corruption ranges from the simple giving of bribes to misappropriation and embezzlement of public funds through the procurement process under the cloak of authority. It is a devastating consequence not only erodes the profitability of organizations and threatens their solvency but also cast doubt on investors’ confidence in the quality of the country’s governance mechanisms. Malaysia has consistently ranked “high” in the corruption index or scale as measured by Transparency International (TI). For 2019, TI’s Corruption Perception Index (CPI) ranks at 51/180 whereas for year 2018 score was at 53/100 ( https://www.transparency.org/en/cpi/2019/index/nzl). For this reason, the increase in the number of reported cases on fraudulent business behavior (i.e., corruption, bribery and fraud) agitates the mind with questions on the motives or reasons for the culprit(s) involvement in the act most especially if the culprit(s) are top-level executives’ level or revered individuals within society. Thus, in order to reduce corruption activities, the government has implemented e-governments to increase transparency and efficiency.

The development of communication and information technology is influential in increasing efficiency or productivity in the business world. In addition, both technologies can improve people’s standard of living globally. ^1^ Governments in several countries have used technology and information to deliver information on government services to the public efficiently. Malaysia has moved on its e-government journey since 1996 with the introduction of the Multimedia Super Corridor (MSC). MSC is an ICT hub hosting more than 900 multinationals, foreign and home-grown Malaysian companies focused on multimedia and communications products, solutions, services, research and development. ^2^ With rapid development and challenges in the digital era, the Malaysian Government created the new Malaysian Digital Economy Blueprint in October 2019 to create a trusted, secure, and ethical digital environment. In addition, the COVID-19 pandemic has accelerated the changes that make the government, businesses, and communities better able to adapt to new ways of daily activities.

In Malaysia Electronic Government Activities Act 2007 (Act680) guided the implementation of electronic government in the public sector activities. The vision of Electronic Government is for government, businesses and citizen for the benefit of Malaysia and its citizens. The objectives more on effectively and efficiently delivering services from the government to the stakeholders, enabling the government to become more responsive and transparent.

For example, Malaysia, under the Malaysian Administrative, Modernization, and Management Planning Unit, launched the eKL project in 2007. eKL seeks to develop public services through an integrated and connected Klang Valley. Based on the theme One Government—Many Agencies, the eKL project sought to integrate service delivery across agencies in order to ensure that services are delivered in a standardized, systematic, and seamless manner. Under eKL, a number of innovations have been introduced. MyBayar is the online payment gateway that offers citizens a convenient and secure way to make online payments to the government. MyForms is the centralized forms directory that makes forms available to citizens and businesses with downloadable and online submission options. In addition, MySMS15888, the short messaging system, is another channel that enables people on the move to stay connected to government services. It provides two-way communication between government agencies and citizens where governmental information, news, and services are made available to mobile phone subscribers anytime and anywhere.

E-government is commonly defined as an information and technology application in providing information as well as connecting citizens with governments. The introduction of e-government is an initiative to streamline the administration of a country. It will make government affairs easier and faster, improving the relationship between people, the private sector, and the government. The government’s digitalization system covers various aspects to ensure the effectiveness of the government’s service delivery system. This includes services related to transportation, immigration, health, and education. This digitalization will reduce over-the-counter services and encourage the use of the internet as a means of delivering government services. Thus, location and time are no longer obstacles for these parties to perform their business. In addition, the global use of technology and the reliability of data in the public service will make it easier for citizens to act and facilitate decision-making. In addition, the reliability of government services will improve relations between the two parties.

E-government implementation could be one of the options for reducing and combating corruption. Technology will reduce the involvement or interaction between public sector service providers and their customers. Excessive interaction between individuals could lead to the existence of elements of injustice and undermine the service provider’s independence. A decision or consideration can be made objectively and fairly with the comprehensive use of technology. In fact, every transaction will be meticulously recorded. A systematic recording system will help to identify red flags or fraud in the organization. However, the smoothness of e-government services will take some time. Government servants and the public must be provided with adequate exposure to adapt to e-government and the e-government system also requires several phases before the system stabilizes.

The Penta-helix model is used in the study of e-government and corruption. The Penta-helix model is a conceptual framework that involves academics, government, industry, non-governmental organizations, and civil society/entrepreneurs/media. It facilitates economic development and pursues innovation and entrepreneurship through collaboration and synergy. ^3^^–^^6^ The interactions between the components in the Penta-helix model facilitate new knowledge and innovation and also create innovation-based economic dynamics. ^7^ Various studies have examined the connection between corruption and e-government. Research on the effect of e-government on corruption highlights that a gap remains in terms of theory and research method that requires further exploration. Several studies on the effect of e-government on corruption were case studies, while others used empirical methods at the macro-level. ^8^ Furthermore, several previous studies have examined the e-government effect on corruption, but without considering other determinants such as political, social, and technological factors. ^9^ Furthermore, the Penta-helix model will help reduce inhibiting factors in implementing the licensing bureaucracy. ^5^ It is practical for problem areas of multi-stakeholders, where they represent a range of a site or problem’s interests. ^10^

This study focuses on developing a model of e-government for fighting corruption in Indonesia and Malaysia by utilizing the penta-helix model, which focuses on the synergy between stakeholders in organizational innovation ^11^ and religiosity. ^12^ Consequently, it is expected that a comprehensive and inclusive e-government model, providing services to the public and empowering public participation in eradicating corruption, will be established. This study selects Indonesia and Malaysia because both are ASEAN countries with similar cultures, beliefs, and understanding. ^13^ In addition, the corruption level in both countries is alarming. ^14^ A big case of corruption occurred in Malaysia. Former Prime Minister Najib Razak is being investigated for his involvement in the misappropriation of funds from the Malaysian strategic company, 1Malaysia Development Berhad (1MDB) which he led. Department US justices allege that $4.5 billion from 1MDB has been abused. Najib Razak was jailed for 12 years after being found guilty of misappropriating $4.5 billion from a subsidiary of the state investment agency SRC International. SRC International is known to be an extension of the 1Malaysia Development Berhad (1MDB) program. 1MDB is a state fund program launched by Najib in 2009 after serving as Prime Minister of Malaysia for four months. ^15^

Meanwhile, the largest corruption case in Indonesia was carried out by the owner of PT Duta Palma Group, Surya Darmadi, which caused a loss of state finances amounting to IDR 78 trillion. The modus operandi is to take over protected forest areas. The former regent of Indragiri Hulu Regency, Raja Thamsir Rachman, granted a location permit and a business permit in a protected forest area of 37,095 hectares in 2004 and 2007 without going through an integrated study and team. The leader of the Duta Palma Group also bribed the Governor of Riau Province with the initials A.M. to make a recommendation that the conversion of protected forest areas managed by five companies owned by Surya Darmadi not become forest areas under the control of the Ministry of Environment and Forestry. The control of oil palm plantations and illegal land tenure generate income for PT Duta Palma Group of IDR 600 billion per month. ^16^

This study was conducted for several reasons. First, the e-government’s application in developing countries involves various challenges. ^17^ Corruption occurs when there is an abuse of power and a lack of religious values. ^12^^,^^18^ Pressure, opportunity, and rationalization can also result in corruption. ^19^ Based on previous studies, it is crucial that this study be undertaken in Indonesia and Malaysia to reveal and share new findings.

Firstly, the governments of both countries have not succeeded in suppressing corruption, which is not worth the cost of eradicating corruption. Furthermore, the ranking of Transparency International highlights that the countries’ positions do not significantly change yearly. ^20^ Therefore, there is a need for e-government transparency through the publication of data on government actions and decisions, the existence of mechanisms to protect whistle-blowers, the maximum punishments for corruptors, and the implementation of information communication and technology.

Secondly, the current system lacks the impact of religiosity and the lack of supervision through the penta-helix dimension, making corruption more crucial. Finally, in practice, e-government is a vulnerable platform and prone to hacking, such as in the e-procurement of goods and services.

## Literature review

### The e-government

E-government implementation in Malaysia began seriously in 1996. In that year, the Multimedia Super Corridor (MSC) project was launched by the government, and e-government was one of the essential components of the flagship application introduced in the MSC. The e-government implementation is intended to increase the community’s productivity and public services quality. In addition, an introduction about e-government will enable people to access services faster, increase competitiveness, and reduce the digital divide in society.

A study by Ramli ^21^ found that as far as e-government implementation challenges are concerned, several enhancements require consideration. For example, there is a low infrastructure technical quality, unstable connectivity, slow speed connection, and limited internet access in certain rural areas. In terms of the structure of legislative, Malaysia has restrictive laws and regulations, outdated regulations, redundant acts, and both security and privacy concerns. Furthermore, Malaysia faced financial constraints of insufficient funds and the necessity on improving the partnership on the public-private. In terms of human infrastructure, it was found that Malaysia did not have an individualistic culture.

Meanwhile, citizens and public officials are slow in adapting to changes. The officials’ public are having difficulty changing in the initial implementation phase, and lack skills as well as expertise, little motivation, and changing management of training. In addition, there is limited integration and coordination, and the issues of federal-state power slow down the implementation of the pace. Issues such as accountability, transparency, and corruption are always associated with the procurement system in Malaysia. This is a major concern of the stakeholders, especially regarding mismanagement and wastage of public money. Contractors and stakeholders demand transparency in the procurement system. Selecting succeeded contractors based on cronyism and personal relationships rather than professional evaluation has become a serious phenomenon. The procurement officers and tender boards were blamed for non-compliance and malpractice with procurement procedures and policies. ^22^

Hamzah *et al*., found that government bureaucracies have created barriers to government services and caused poor accountability and transparency, thus, discouraging citizens’ dealings with government agencies. ^23^ Therefore, society must change and be open to technology. The e-government must quickly implemented so that the citizens and government can make a connection to the virtual space that created and strengthened through the leadership that support governance framework. ^23^ The government acknowledges that technology enables various social problems, including corruption, to be addressed and will continue to find the best mechanisms by using the latest technologies to combat corruption. As stated by Ramli, a positive connection between corruption and e-government when society reaches high level of economic growth and development. ^24^ This is also supported by Rustiarini and Sudiartana, where different organizational and external factors change the e-government functions’ effectiveness in fighting corruption. ^25^

In Malaysia, the National Anti-Corruption Plan (NACP) 2019–2023 states that technology can be used to prevent corruption where complaints are obtained through open public sources with transparent processes. The electronic process has seen procurement and payments activities run more transparently through digital transactions, reducing the risk of corruption. ^24^ The latest technology, which indirectly creates a safer audit trail, is more reliable and trusted by stakeholders. In addition, information technology facilitates access to information and promotes transparency and openness that contribute to fair and equitable competition for the government and the market. Therefore, through the digitalization of the civil service, bureaucracy and the risk of corruption and government costs can be reduced. ^26^

For Indonesia, attempts have been made to eradicate corruption. The Indonesian government focuses more on reporting the application of electronic-based state finances, starting from local governments to the central government and maximizing the institution of an independent corruption eradication commission. In addition, the implementation of e-audit in financial audit institutions aims to examine the use of state finances more quickly and accurately. Therefore, implementing e-government and e-audit in every part of the government office can reduce acts of fraud and corruption in government institutions.

### Anti-corruption strategy

One of the strategies to prevent acts of corruption is to implement e-government. A study by Alhammadi and Alhadramy highlights the actions to prevent corruption through the implementation of e-government which includes ^27^:

a) The use of services undertaken online will increase efficiency and reduce discretionary abuse and other corruption’s opportunities.
b) The function of e-government developed through the government must be complemented by the development of institutions, laws, and practices that protect whistle-blowers and provide disincentives and laws against perpetrators of corruption.
c) E-government will reduce discretionary power and control the operations of government officials. In addition, activities undertaken by the public will be tracked, controlled, and stored for future reference.
d) E-government tools track various illegal events and actions and proactively detect suspicious behavior before any crime is committed. Thus, officials are more careful in trying to engage in corrupt behaviors since transactions are documented and easily traceable.
e) Government information is available online. Citizens can simply access and connect to the government services and information anywhere, anytime, and through various channels. Thus, it helps citizens provide proper documentation when lodging their complaints against corrupt practices.

### Penta-helix model

The penta-helix model is often used in fields related to socio-economic development. The model contains five important elements namely (i) public, (ii) private, (iii) academia, (iv) society, and (v) entrepreneurs. These five elements support the achievement of the economic development level. They have roles and functions in the development of society. The penta-helix model helps to improve the economy through innovation and entrepreneurship, and collaboration and synergy among the players. ^3^^–^^6^ They are agents of local economic change and promote sustainability in economic activities. Therefore, the economic development of an area will be determined by their commitment and contribution.

In achieving the socio-economic development goals, these five parties must ensure that the goals are running smoothly. Therefore, close cooperation and understanding must be established. The importance of a synergistic relationship between the five components in the penta-helix model is vital, where the interaction between these components will result in innovation and knowledge and create dynamics of an innovation-based economy. ^8^^,^^28^ Such synergistic relationships include ensuring that economic activities are conducted ethically by avoiding activities that contribute to elements of corruption in business. This is because these elements are interdependent in the business ecosystem, and corrupt activities can be avoided if each of these elements acts seriously to address them. Therefore, each key player must have a sense of responsibility and work together so that the relationship established contributes to fair and equitable economic development.

### Fraud triangle dimension

Fraud is defined in various ways. However, the focus is a person’s deceitful behavior that is designed to benefit oneself by misrepresentation. ^29^ A fraudulent financial statement is a designed act that impacts in a misstatement material in the financial statements. Meanwhile, Sihombing and Rahardj ^30^ said that fraudulent financial reports are intentionally produced in financial statements that are not in conformance with commonly accepted principles of accounting. Fraudulent reports are material and can influence the decisions of attentive parties. Compatible with the Australian Audit Standard in US and European, a fraudulence financial report is an premeditated misstatement, as well as the omission of amounts or a leak in financial statements to mislead the users of these statements. ^31^

**Figure 1.**
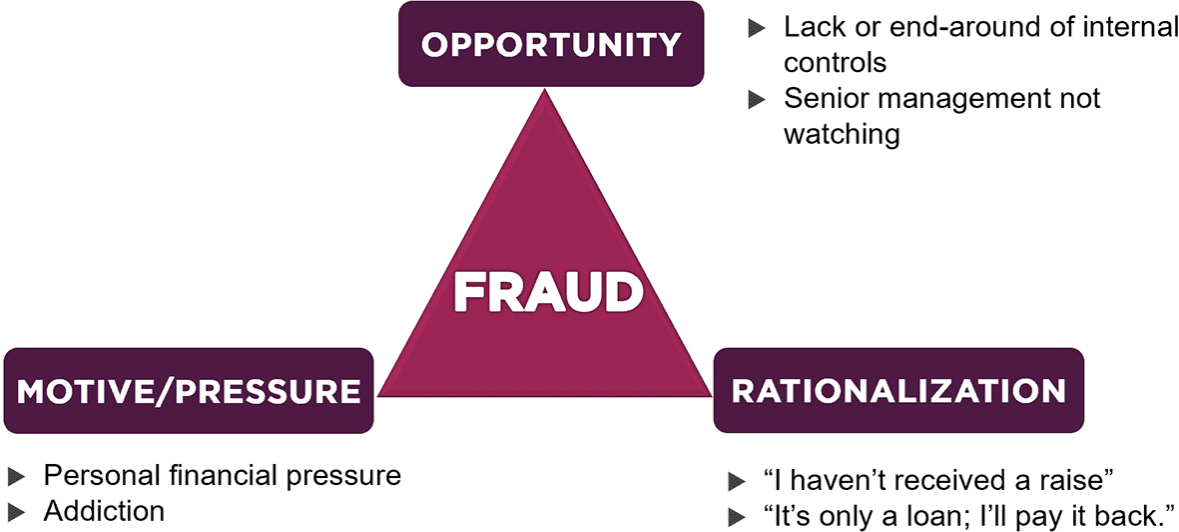
Fraud Triangle Theory.

The theory of fraud was first expressed by Skousen *et al*., ^19^ which consisted of insurmountable financial issues, opportunities to commit violations and rationalization by offenders. Over time, Cressey’s hypothesis developed rapidly and became known as “the fraud triangle”. Details about the fraud triangle are depicted in Figure 1.

### Religiosity

Individual behavior patterns are affected by a set of values consisting of intrinsic and extrinsic values. One aspect of these values is the religious value. ^32^ Religious values are those that inspire self-confidence and faith in an individual. These values guide an individual to act obediently and honestly in dealing with daily activities. ^33^ A person’s religious values can affect self-control and reduce deviant behavior. ^34^^,^^35^ A study found that religiosity positively correlates with risk-averse actions. In fact, in the case of some religions and religious people tend to avoid risky actions such as corruption. ^36^

However, there is another perception of corruption based on religiosity and social trust. The view rests on the belief that a higher divine power will punish individuals for their evil deeds in this lifetime or next. With these beliefs, people will not engage in corrupt behavior, and there is no need to monitor the corrupt behaviors of others, ^37^^,^^38^ including in government institutions. ^39^ In addition, trust in other people and law enforcement systems may reduce the demand for accountability of actions. Furthermore, fewer actions are required to monitor the elected officials by having more religious government officials. ^40^

A person’s level of faith can indirectly influence a politician into political corruption. Religious assistance is shown both empirically and theoretically to increase political aid ^41^ and civic commitment. ^42^^,^^43^ However, the motives behind such commitment could be due to rent-seeking, which increases political corruption. In addition, there are other economic motivations for those who are religious: religion can add to an individual’s moral character, as a principal value in transaction of the marketplace. ^44^ Futhermore, people in the same organization can be more tolerant of corrupt behavior carried out by others who are part of the same organization’. ^45^

Investigations of the relationship between religiosity and corruption hace found different results. ^46^ Research in the United States shows that there is no relationship between the level of corruption and religiosity. ^46^ Futhermore, in tests done globally, a negative relationship between the level of corruption and religiosity has been founf. ^47^ However, this study did not control certain standard variables associated with corruption, such as per-capita GDP, cultural colonialism or the effectiveness of the legal system. A study by Yahya *et al.,* ^48^ found that employee religiosity and organizational culture are essential in determining the corruption millennial employment. This finding suggests that it is important for management to emphasize the importance of a positive inconclusive organizational culture and religious awareness among employees. A study by Barbier *et al.,* ^49^ found a negative relationship between individuals’ religiosity and their responses to what is “justifiability of corruption,” thus, suggesting there is no aggregate government corruption.

Corruption is detrimental to the overall well-being of a country’s economic system. While legal enforcement systems are available in the majority of countries, corruption still exists. Many researchers have analyzed the factors that correlate with corruption in different countrie and one of the main factors that influence the level of corruption is religiosity. ^50^ However, previous research has had very little explicit focus on the difference between religion and religiosity. A study by Gokcekus and Ekici uses data from various countries and experiments with varying measures of religiosity. ^50^ The empirical proof indicates that religiosity, rather than a religious affiliation, influences corruption levels. Societies that are more religious typically have higher levels of corruption, despite the country’s religious affiliation.

## Hypothesis development

### E-government corruption effect

The primary cause of corruption in the government sector is the asymmetry of information between the government and public. Consequently, this can result in bureaucrats setting ambiguous standards of government service. This facilitates bribery through secret contacts with the public, and the public is more likely to respond to the agreement. ^51^ E-government can reduce corruption because it can control corruption through transparency, ^52^ disclosure, and the monitoring of government administration using technology. ^53^^,^^54^ Research reveals that e-government is beneficial in reducing corruption. ^55^^–^^59^ In terms of the use of e-government in the community, more attention is given to the transparency, objectivity, publication of government budget information, and feedback related to services and policies. ^60^ The solutions offered by the e-government expect sophistication from a technical perspective. Furthermore, they require a complete reorientation of the bureaucracy, especially, the awareness to conduct duties and functions to be neutral and genuinely in the mission of public service. ^60^

Therefore, e-government is essential in preventing corruption if the maturity of e-government implementation involves public participation in information, public participation in supervising the government, and actions from government officials that substantially increase transparency. ^61^^–^^62^ Furthermore, the synergy between academics, businesses, the community, media, NGOs, and governments are elements of the penta-helix. Therefore, this study hypothesized that:

*H _1_: E-governance has a positive effect on corruption.*

### E-government, religiosity and corruption

People with intrinsic religiosity can be defined as people having reached a certain level of personal integrity and maturity. They have made an unconditional commitment toward religion and make decisions independently. Religious orientation becomes an inseparable part of one’s religious maturity. ^33^ Specifically, religiosity refers to a person’s belief, practice, experience, identity, and attitude. ^63^ For Muslims, religiosity means adherence to God and desire to be a good person. ^18^

Research by McGee *et al.,* ^64^ and Shadabi ^65^ has highlighted that religion could influence human behavior and individual decision making. A study found that religiosity can moderate the relationship between corruption and e-government because a person’s reaction is dependant on their beliefs, values and responsibilities. ^33^ In addition, Said *et al*., ^66^ identified that religiosity is significantly give impact to asset misappropriation. Higher religiosity in a person lowers the probability of asset misappropriation. Therefore, individuals with a high level of religiosity would attempt to avoid deviant actions. ^67^ Therefore, a religious background and knowledge instilled in an individual can help reduce their level of corruption because they can control themselves in facing normal situations and irregularities within the workplace. ^34^ Hence, religiosity can prevent individuals from committing fraud and corruption. ^33^ Therefore, based on previous studies, this study hypothesized that:

*H _2_: Religiosity has a moderating effect on the link between E-government and corruption*

**Figure 2.**
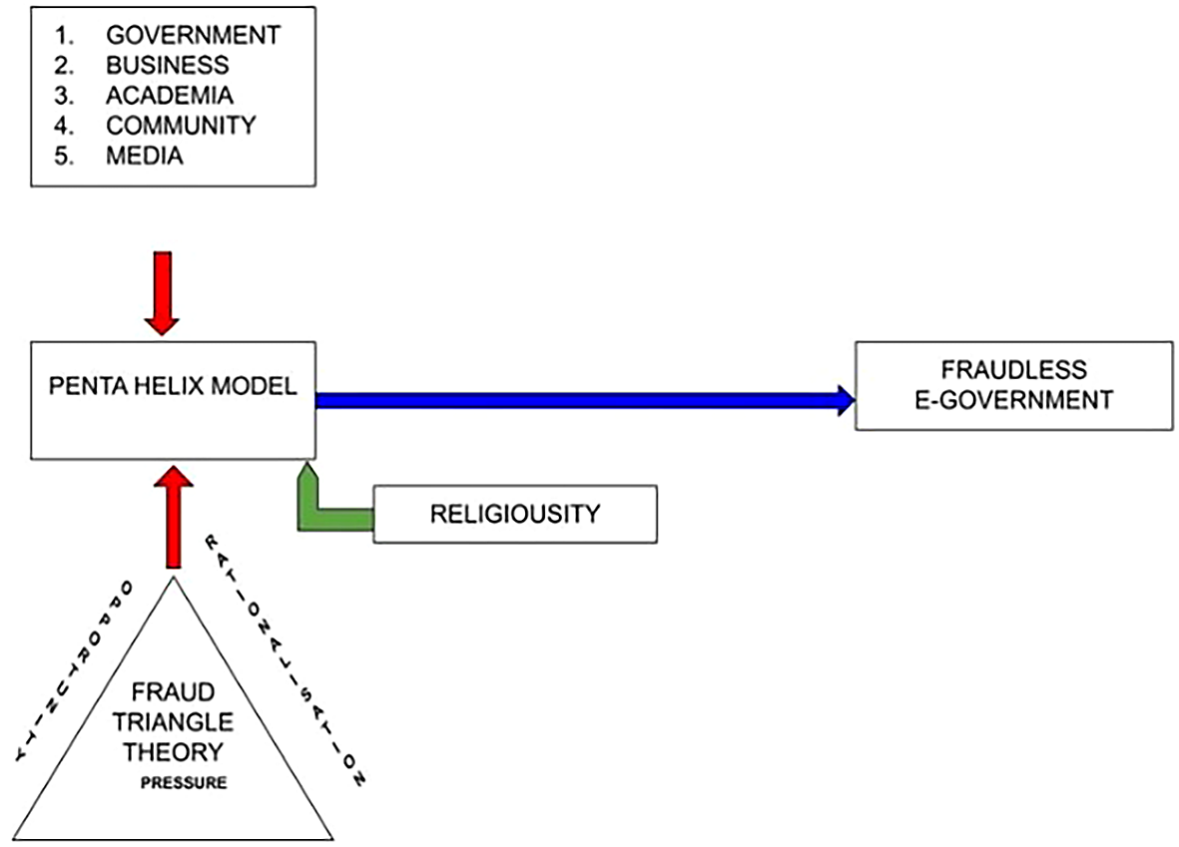
Research Model of Corruption.

The penta-helix model is used in this diagram to predict the forms, causes, and efforts that prevent corruption. This study involved five group of participants from both the Indonesian and Malaysian Governments, businesses, community, academia, and the media. The moderating of religiosity highlights how humans behave and comply with rules and regulations. This determines how people react when facing the risk of corruption and fraud. ^61^

The fraud triangle theory in the diagram indicates that “rationalization” is a perspective related to moral and psychological elements, which are essential in identifying the causes unethical behavior. ^68^ Religiosity can be a “zero tolerance” policy toward unethical behavior and can be a preventive factor when they consider undertaking unethical behavior.

## Methods

### Ethics

This study was accepted and approved by the Universitas Islam Bandung Ethical Clearance Committee (no. 719/B.04/Bak-k/XII/2020). In addition, written, informed consent was obtained from participants. The variable utilized in this study is e-government which is divided into three sections (3) –the Government to the Citizen (G2C), the Government to the Business (G2B), and the Government to the Government (G2G). Meanwhile, the penta-helix was measured by elements such as government officials, academia, media, NGOs, the community, and businesspeople. The religiosity variable was measured based on ideology, practice, experience, knowledge, and consequences. Finally, corruption was measured using opportunity, pressure, and rationalization. The variables are operationalized in Table 1:

**Table 1.** Variable's Measurement.

Variable	Dimension	Indicator	Measurement
E-Government ^69^	G2C	Public information	- Clarity - On-time - Easiness
	G2B	E-procurement	- Availability - Ownership
	G2G	Provide information	- Solve problem - Accessible - Feedback
Penta-helix ^3^^–^^6^	NGO	- Involvement - Role - Participation	- Clarity - On-time - Easiness
	Businessmen	- Fair competition - E-procurement	- Clarity - On-time - Easiness
	Government officials	- Function - Action - Evaluation	- Clarity - On-time - Speed
	Academia	- Role - Involvement - Participation	- Clarity - On-time - Easiness
	Media	- Educate - Controlling - Supervising	- Clarity - On-time - Easiness
Religiosity ^34^^,^^35^	Ideological	- Belief	- Clarity - Easiness
	Practice	- Worship - Convenience	- Clarity - Easiness
	Experience	- Activity	- Clarity - Easiness
	Knowledge	- Law understanding	- Clarity - Easiness
	Consequence	- Obedience - Responsibility	- Clarity - Easiness
Corruption ^70^	Policy	- Report	- Clarity - Firmness
	Ethical	- Checking	- Available - Intention

### Sample selection

This study utilized a sample of 240 respondents from Malaysia and Indonesia between March to May 2021. These countries were selected because they share similar cultures and beliefs. In addition, the corruption level in both countries is alarming. The respondents represented those in the petha-helix model: government officials, academics, those working in the media, NGOs, the community, and business people, in Malaysia and Indonesia. Respondents included government auditors, university lecturers, by news employees, NGO employees in the social sector, community employees in the economic and social sector; and business people at the Ministry of Micro, Small and Medium Enterprises (MSME) level. The sample was selected based on purposive sampling. ^71^ The respondent selection criteria was based on their job description and experience or daily use of e-government services. Respondents who had never used e-government were not included in the sample criteria. The respondents were contacted by the research team, using established networks of friends, office colleagues, families, and alumnus to recruit participants. The researchers then approached the respondents through telephone calls, email, or WhatsApp messages. After explaining the aims of the study to the respondents and receiving their consent, a questionnaire on Google Forms was sent either through email or WhatsApp.

### Data analysis techniques

This study used a quantitative approach for the data collection procedure. Before this questionnaire was distributed to respondents, it was tested on 30 respondents, using a cross sectional method through focus group discussions in February 2021. Based on this pilot project of 30 respondents, the results showed that the instrument the questionnaire was valid and reliable.

After receiving comments and refining the questions from the 30 respondents in the pilot study, the revised questionnaire was distributed to 240 new respondents via emails and WhatsApp message using the Google Form platform, between March to May 2021. The questionnaire was divided into five sections. Section A: respondent profile; Section B: The implementation of e-government in local authority; Section C: perspectives related to fraud triangle dimensions; Section D: beliefs and religiosity; and Section E: strategy to combat corruption.

The descriptive and inferential non-parametric statistics (part of inferential statistics) were used in analyzing the data. The SPSS v.25 was used to analyze the data for Malaysia and Indonesia datasets. Subsequently, this study conducted a reliability and validity analysis to assure the stability of the data. The estimated r-value was compared to the tabled r-value in determining the validity value. The validity test was deemed passed if the calculated r-value was larger than the tabled r-value. While the reliability test was conducted using Cronbach’s Alpha with criteria ranging from 0.80 to 0.88.

## Results

### Demographic profile

Four demographic information were gathered from the 240 respondents from Malaysia and Indonesia— gender, education, computer abilities, and daily computer usage. First, there were more female than male participants from both countries in this study (Indonesia = 53%; Malaysia = 50%). Second, the average age is 36–45 years old (37% for Indonesia and 47% for Malaysia). Third, the respondents’ average educational level is an undergraduate degree (43% in Malaysia and 42.5% in Indonesia). In addition, this study shows no difference in terms of computer skills between Indonesia (79%) and Malaysia (80%). Finally, the average daily computer usage is six (6) hours per day (36% in Indonesia and 47.5% in Malaysia). Respondents’ complete information of demographic is displayed in Table 2:

**Table 2.** Demographic data.

	Indonesia	Malaysia
**Gender**		
Male	47%	50%
Female	53%	50%
**Age**		
25–35	27%	28%
36–45	37%	42%
46–55	27.50%	27.50%
Over 55	9.10%	2.50%
**Education Level**		
Diploma	12.50%	17%
Undergraduate	42.50%	43%
Master	21%	17.50%
Doctor	14%	12.50%
Other	10%	10%
**Level of Computer Skills**		
Beginner	5%	5%
Intermediate	79%	80%
Expert	16%	15%
**Computer Usage/Days**		
1 Hour	19%	19%
6 Hour	36%	47.50%
15 Hour	30%	29%
Over 20 Hours	15%	5%

### Test of validity and reliability

The results from the test of validity and reliability show that all items have an r-count value larger than the interpolated r-table (n = 240, = 5% = 0.133). All items are declared valid and reliable based on the validity and reliability testing results. Furthermore, the variables analyzed have a positive and notable effect for Indonesia and Malaysia since the p-value is below 0.133, except for the religiosity variable, which is not too significant because larger p-value (0.162), as shown in Table 3.

**Table 3.** Regression weight structural equation.

	Indonesia				Malaysia			
	Estimate	S.E.	C.R.	P	Estimate	S.E.	C.R.	P
E-government ➔ Corruption	0.297	0.168	1.767	0.077	0.058	0.080	0.722	0.034
Religiosity ➔ Corruption	−0.677	0.502	−1.348	0.162	0.112	0.192	0.577	***
E-government* Religiosity ➔ Corruption	0.064	0.041	1.565	***	0.065	0.028	2.324	***

Table 4 highlights that the effect of e-government on corruption in Indonesia is 0.132, whereas the effect of religiosity on corruption is negative (−0.276). Nevertheless, the presence of religiosity as a moderator can boost the effect of the e-government on the corruption by 0.321. However, the effect of the e-government on the corruption in Malaysia is relatively minimal at 0.066—religiosity is only 0.053, and religion as a moderator is 0.209. Therefore, the impact of religiosity toward e-government corruption is minimal. Therefore, in comparing the moderating effect of religiosity in both countries, it appears that religiosity significantly strengthens the influence of the e-government on the corruption in Indonesia more effectively than in Malaysia. The following graph depicts the number of e-governments on corruption in Indonesia and Malaysia and its moderating effect. Interestingly, these findings provide evidence that religiosity is influential in combating acts of corruption in government agencies. Thus, the regulatory bodies and the governments of both countries must be aware that religiosity may influence the behavior of the officials.

**Table 4.** Standardized Regression Weights: (Group number 1—Default model).

	Indonesia	Malaysia
Variables	Estimate	Estimate
E-government ➔ Corruption	.132	.066
Religiosity ➔ Corruption	-.276	.053
E-government* Religiosity ➔ Corruption	.321	.209

**Figure 3.**
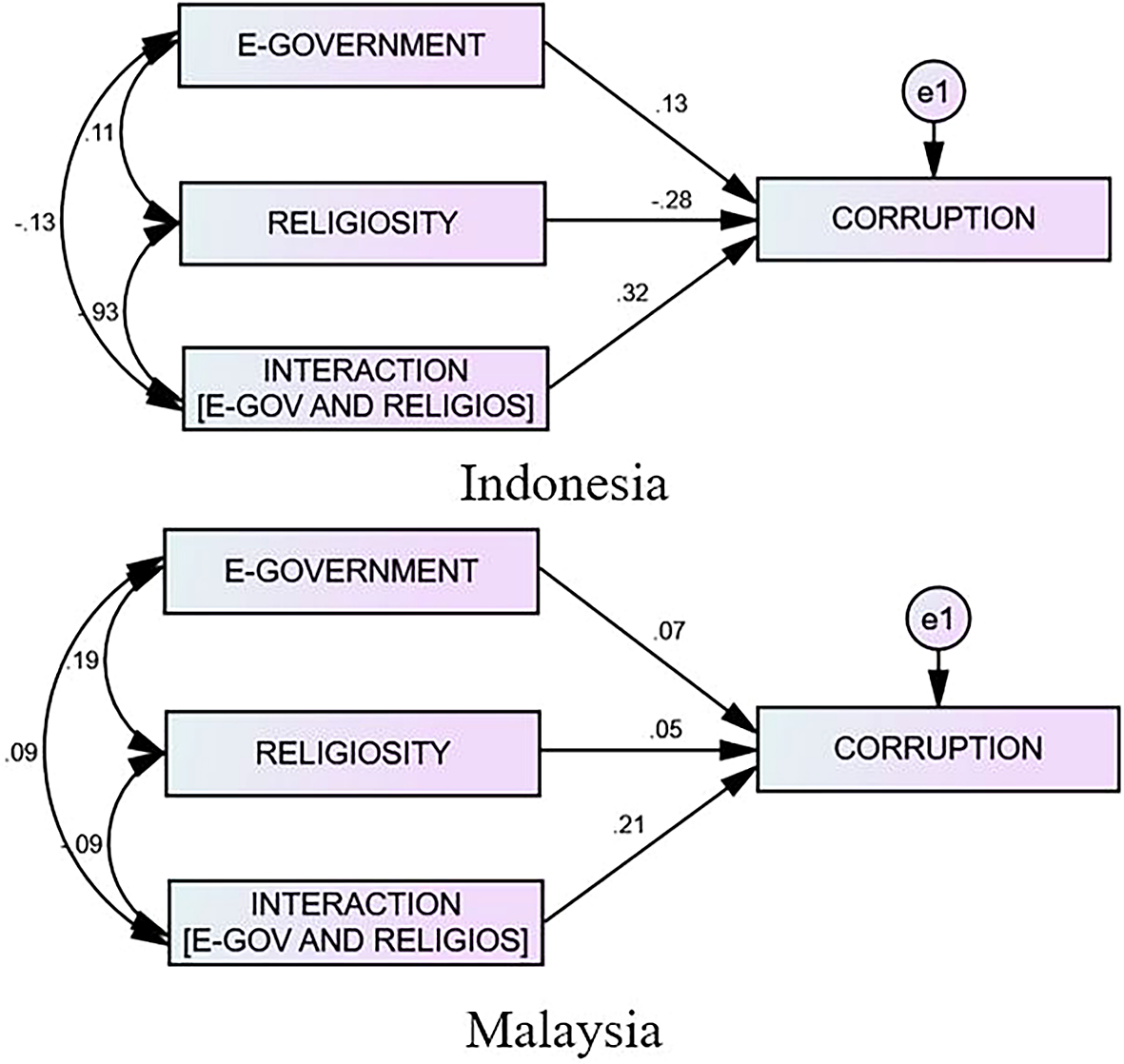
The Result of the Interaction Model Between Indonesia and Malaysia.

## Discussion

The results of Indonesia’s e-government rollout reveal that e-government implementation had an effective and significant effect on reducing corruption, as indicated in Table 4 (0.132). According to Sosiawan, ^72^ e-government deployment is considered a “dare-dare” project or merely a fleeting trend. Therefore, all government policies, legislations, and practices, including the use of e-government, impact corruption. This implies that public transparency is required. This finding supports Nam’s ^52^ assertion that e-government can minimize corruption since it has the potential to control it through openness. This conclusion contradicts the findings that claim that the e-government negatively impacts corruption. ^55^^–^^59^^,^^62^

The findings suggest that the majority of e-government providers, including government and non-government organizations, are still “secure” and “comfortable” with website owners and are no longer concerned with optimizing e-government use. However, there are hints that e-government is simply a project to “sell” communication and information technologies, both hardware and software, to “traders.” This indicates that one’s beliefs and religion do not become a barrier against acts of corruption, but that religion is utilized as a tool and justification for corruption by an official. According to the findings, based on the penta-helix model, government involvement can help create a clean, committed, and high-integrity work environment. NGOs can promote improvements in government officials’ behavior and the elimination of official corruption. Therefore, while academia may help analyze research data and facts for the government to consider when making policies and decisions, data and news can be communicated to the public in a timely, accurate, and transparent manner, and businesspeople can follow and maintain good business ethics in compliance with the laws. Meanwhile, religion is a moderator that can boost the influence of e-government on corruption (0.321). It can positively reduce the acts of corruption, particularly the religiosity variable, which will become more important in reducing corruption in Indonesia. E-government within Malaysia shows a positive impact on both corruption (0.066) and religiosity (0.053), as displayed in Table 4. The results also highlight that religiosity moderates (0.209) and strengthens the impact of e-government on corruption in Malaysia.

In summary, using the penta-helix model, the NGOs, government, academia, businesspeople, media, and the community can be leveraged to boost anti-corruption efforts. Similarly, religious qualities such as belief, kindness, and trust can support anti-corruption efforts. ^68^ The result on religiosity aligns with previous studies, showing that religiosity can strengthen the efforts to eradicate corruption. ^33^^,^^67^ Thus, religion can be employed as a moderating variable in both Indonesia and Malaysia to improve the influence of e-government on corruption, reduce pressure from other parties, close the chances for corruption, and reduce the problem of rationalization.

**Figure 4.**
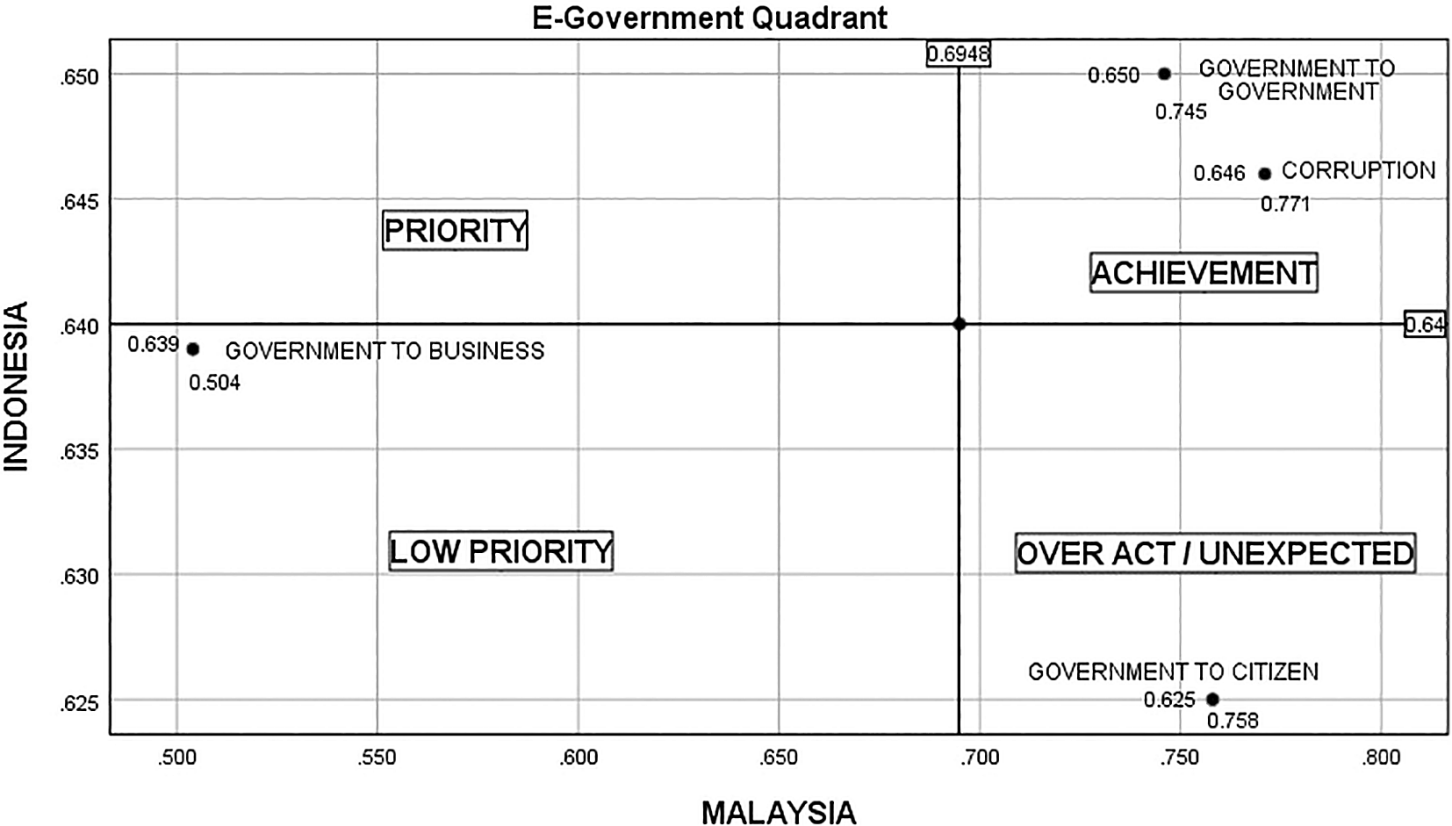
Quadrant of E-government.

There are four quadrants in Figure 4, representing four situations—low priority, unexpected, priority, and achievement. In Indonesia, there are still many cases of bribery in project tenders, unscrupulous officials marking up project values, businesspeople and officials flirting with the acquisition of government projects, the greedy nature of unscrupulous officials and businesspeople, and a lack of fear of God for their actions. Therefore, G2B conditions in Indonesia are higher (0.639) than in Malaysia (0.504). Meanwhile, in Malaysia, the cases of bribes related to procurement are still high, as reported in the National Anti-Corruption Plan. However, the state of G2C in Indonesia is lower (0.625) than in Malaysia (0.758) due to the lack of community initiative and apathy in supervising government and industry projects, and boredom with government promises to eradicate corruption that has never been fulfilled. ^66^ Both countries reported a high value for G2G, which is above the average score in attempts to eradicate corruption. However, based on the G2G implementation (Indonesia, 0.650; Malaysia, 0.745), Malaysia’s level of eradication is significantly greater than Indonesia’s.

Consequently, both countries aim to have a high level of corruption prevention and eradication by fostering good governance and growing G2B and G2C engagements. For both countries, the influence of e-government adjusted by religion has a favorable and significant impact. According to the laws, anyone with a high level of religiosity can ensure that e-government projects are completed on-time while avoiding corruption. However, because poor religious comprehension is reflected in daily actions, weak faith is readily seduced to commit corruption, worship obedience can be a ruse, and religious regulations that should be understood are ignored.

Therefore, the e-government system is a strategy that can be used in Indonesia and Malaysia to prevent corruption. Strengthening the understanding of religious beliefs and elements of penta-helix will help both countries to have better transparency and a strong governance system. With reference to the penta-helix model, the government officials’ strategy is to react truthfully and be transparent in their everyday operations. The role of the media is to be transparent by sharing the latest news and activities of influential people or political elites involved in corruption. Meanwhile, businesspeople aim to encourage business actors to use the e-government platform to perform government-related commercial activities. The role of the NGOs is to oversee the monitoring and the implementation of the e-government process. Meanwhile, academia is encouraged to conduct further research on the prevention of corruption. The most important element is to encourage government officials to improve and increase their religiosity to strengthen their faith. Consequently, they will be less likely to commit corruption.

Based on the results of the above analysis, the preventive strategy for the Indonesian state is to make project tender procedures electronically transparent and clear without a lot of bureaucracy, and to increase community involvement to oversee the process of open project auctions, oversee procurement and project implementation (G2B). By creating a clean and transparent bureaucracy for the government and industry, it will reduce and prevent corruption.

As for Malaysia, the corruption prevention strategy is carried out by creating a sustainable communication model between the government and the community (G2C), building public information center to report and complain about alleged corruption. But the most important thing for both countries (Indonesia and Malaysia) is to form a religious character towards employees, law enforcers, and entrepreneurs by continuously reminding and emphasizing the importance of honesty and fear of God. and include the cost of zakah and shodaqoh in each project value.

## Conclusion

This study found that the implementation of the e-government has positive and significant effects on efforts to prevent corruption in Indonesia and Malaysia. In addition, it proved that religiosity is essential in increasing the influence of e-government in preventing corruption by including elements of the penta-helix. The findings confirmed the objective of the study that penta-helix and religiosity are vital elements to be applied in the government of both countries, especially in the e-government settings. However, this study is limited in that it only considers two ASEAN countries. Therefore, future studies may consider a more extensive dataset which may include other ASEAN countries such as Singapore, Thailand, and Brunei. Moreover, this study only considers the fraud triangle theory. However, further research can apply the “law and regulations” variables, as mentioned in the development of the penta-helix model, called the hexa-helix model, so as to produce stronger results.

## Data availability

### Underlying data

Figshare: Dataset of Questionnaire Result from the respondents of Penta-helix model of e-government in combating corruption, https://doi.org/10.6084/m9.figshare.19643325.v1. ^73^

This project contains the following underlying data:

• Dataset of Questionnaire Result from the respondents of Penta-helix.csv

### Extended data

Figshare: List of questions and descriptions of questionnaire of the Penta-helix model of e-government in combating corruption in emerging markets: Religiosity as a moderating, https://doi.org/10.6084/m9.figshare.19643607.v1. ^74^

This project contains the following extended data:

• List of questions and descriptions of questionnaire of the penta-helix model.csv

Figshare: The Respondent characteristics of the penta-helix model of e-government in combating corruption in emerging markets: Religiosity as a moderating role, https://doi.org/10.6084/m9.figshare.19643340.v1. ^75^

This project contains the following extended data:

• The Respondent characteristics of the penta-helix model of e-government.csv

Data are available under the terms of the Creative Commons Attribution 4.0 International license (CC-BY 4.0).
