# Supplementary material for: Penta-Helix Model of E-Government in Combating Corruption in Indonesia and Malaysia: The Moderating Effect of Religiosity
Source: F1000Res. 2022 Nov 29;11:932. Originally published 2022 Aug 15. [Version 3] doi: 10.12688/f1000research.121746.3 (PMC9709345; doi:10.12688/f1000research.121746.3)
Supplement: Peer review history

# Peer review: Reviewer response for version 3

**DOI:** 10.5256/f1000research.141527.r156859

**Published:** 2022-11-29

**Authors:** Irma Tyasari

**Affiliations:** 1 Faculty of Business and Economics, Universitas Kanjuruhan Malang, Malang, Indonesia

The authors have addressed all the comments, both in the introduction and the conclusion sections. Their manuscript has been improved.

Is the work clearly and accurately presented and does it cite the current literature?

Yes

If applicable, is the statistical analysis and its interpretation appropriate?

Yes

Are all the source data underlying the results available to ensure full reproducibility?

Yes

Is the study design appropriate and is the work technically sound?

Yes

Are the conclusions drawn adequately supported by the results?

Yes

Are sufficient details of methods and analysis provided to allow replication by others?

Yes

Reviewer Expertise:

Auditing, financial reporting, financial management, leadership, quality control system, corporate governance, risk management

I confirm that I have read this submission and believe that I have an appropriate level of expertise to confirm that it is of an acceptable scientific standard.

# Peer review: Reviewer response for version 2

**DOI:** 10.5256/f1000research.138689.r155245

**Published:** 2022-11-22

**Authors:** Irma Tyasari

**Affiliations:** 1 Faculty of Business and Economics, Universitas Kanjuruhan Malang, Malang, Indonesia

This is an interesting and contemporary topic by addressing e-government and corruption in two ASEAN countries. 

 Comments: 

1. The paper discusses about the corruption and e-government in Malaysia and Indonesia. However, the author may consider to establish the context underlying the study, based on these two countries. Since, the introduction focuses more on the Malaysia phenomenon. 
2. In the discussion or conclusion, the author may add and provide the finding that can be a good and substantial contribution to each home country specifically based on the data analyzed, not only mentioned in general like " the study that penta-helix and religiosity are vital elements to be applied in the government of both countries". 

Is the work clearly and accurately presented and does it cite the current literature?

Yes

If applicable, is the statistical analysis and its interpretation appropriate?

Yes

Are all the source data underlying the results available to ensure full reproducibility?

Yes

Is the study design appropriate and is the work technically sound?

Yes

Are the conclusions drawn adequately supported by the results?

Yes

Are sufficient details of methods and analysis provided to allow replication by others?

Yes

Reviewer Expertise:

Auditing, financial reporting, financial management, leadership, quality control system, corporate governance, risk management

I confirm that I have read this submission and believe that I have an appropriate level of expertise to confirm that it is of an acceptable scientific standard, however I have significant reservations, as outlined above.

# Response

**Published:** 2022-11-24

**Authors:** Pupung Purnamasari

**Affiliations:** Universitas Islam Bandung, Indonesia

Thank you for the review that has been given to our article. Furthermore, this article has been revised in the introduction section adding studies in Indonesia. In the concluding section, the authors have provided additional information regarding findings that can be a good and substantial contribution to the countries being analyzed, namely Indonesia and Malaysia.

# Peer review: Reviewer response for version 2

**DOI:** 10.5256/f1000research.138689.r154862

**Published:** 2022-11-14

**Authors:** Hussaini Bala

**Affiliations:** 1 Department of Accounting, Faculty of Administrative Sciences and Economics, Tishk International University, Irbid, Iraq

The authors have made a significant improvement on the manuscript by effecting all the corrections required.

Is the work clearly and accurately presented and does it cite the current literature?

Yes

If applicable, is the statistical analysis and its interpretation appropriate?

Yes

Are all the source data underlying the results available to ensure full reproducibility?

Yes

Is the study design appropriate and is the work technically sound?

Yes

Are the conclusions drawn adequately supported by the results?

Yes

Are sufficient details of methods and analysis provided to allow replication by others?

Yes

Reviewer Expertise:

Financial reporting, auditing, corporate governance, sustainability reporting, and taxation, financial management, economic growth.

I confirm that I have read this submission and believe that I have an appropriate level of expertise to confirm that it is of an acceptable scientific standard.

# Peer review: Reviewer response for version 1

**DOI:** 10.5256/f1000research.133645.r147536

**Published:** 2022-09-05

**Authors:** Hussaini Bala

**Affiliations:** 1 Department of Accounting, Faculty of Administrative Sciences and Economics, Tishk International University, Irbid, Iraq

The authors have done well by addressing an interesting topic ‘Penta-Helix Model of E-Government in Combating Corruption in Indonesia and Malaysia: Religiosity as a Moderating Role. However, I have the following comments that can be used to enhance the quality of the manuscript.

**Major Comments**

1. The focus of the paper is corruption in Indonesia and Malaysia. However, the introduction majorly concentrates on E-Government. Therefore, there is need for the authors to highlight the prevalence of corruption in Indonesia and Malaysia and how E-Government may help in combating the corruption.
2. There is need to amend the research framework to reflect the title of the paper. This is because the current framework in the paper displays E-government as a dependent variable.

**Minor comments**

1. You may amend the topic to ‘Penta-Helix Model of E-Government in Combating Corruption in Indonesia and Malaysia: The Moderating effect of Religiosity.'
2. In the literature review section, change the title ‘E-government corruption effect moderated by religiosity’ to ‘E-government, religiosity and corruption.' Or 'Influence of religiosity on the link between E-government and corruption’.'
3. 'H2: E-government has a positive effect on corruption moderated by religiosity' should be changed to ‘H2: Religiosity has a moderating effect on the link between E-government and corruption.'

Is the work clearly and accurately presented and does it cite the current literature?

Yes

If applicable, is the statistical analysis and its interpretation appropriate?

Yes

Are all the source data underlying the results available to ensure full reproducibility?

Yes

Is the study design appropriate and is the work technically sound?

Yes

Are the conclusions drawn adequately supported by the results?

Yes

Are sufficient details of methods and analysis provided to allow replication by others?

Yes

Reviewer Expertise:

Financial reporting, auditing, corporate governance, sustainability reporting, and taxation, financial management, economic growth

I confirm that I have read this submission and believe that I have an appropriate level of expertise to confirm that it is of an acceptable scientific standard, however I have significant reservations, as outlined above.
